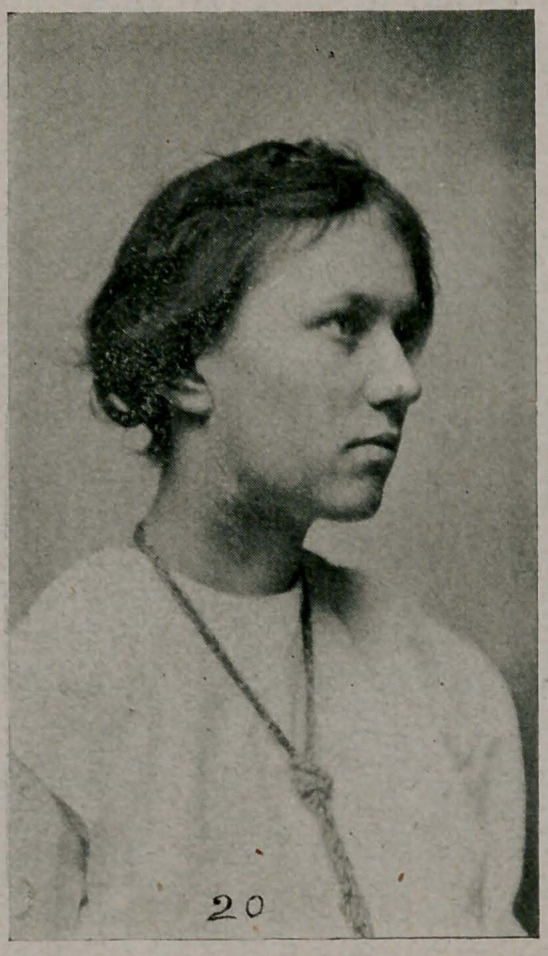# Types and Symptoms of Goiter and Hyperthyroidism

**Published:** 1914-12

**Authors:** 


**Types and Symptoms of Goiter and Hyperthyroidism.** Addison G. Brenizer, A. B., M. D., Charlotte, N. C. The Old Dominion Journal of Medicine and Surgery. (Cuts by courtesy of author and editor.)

Case I. (Photos 1 and 2). A man of fifty with an abscess formed in the thyroid gland, developing in the course of six days, and accompanying a severe influenzal laryngitis and bronchitis. The abscess ruptured into the trachea on the 10th day and the tumor subsided on the 12th day (photo 2).

1
2
3
4
5
6

7
8
9
10
11
12

Case II. (Photo 3). A young woman of twenty-six, who gave the history of numerous attacks of tonsilitis, accompanied by glands in the neck. Her eyes became very prominent and the front of her neck swollen (thryoiditis with over-secretion). The swollen tonsils, the glands, and the swelling over the neck subsided and the eyeballs receded; the lids remained dropped down over the eyes (destruction of thyroid tissue; sympathetic paralysis on both sides.) The patient was benefited by thyroid extract, but the lids remained dropped. An operation for suspension, modified after the Hess technic was performed (Drs. Cauthen, Allen and Brenizer).

Case VII. (Photo 4.) Middle aged woman, with a very vascular goiter. The tortuous veins can be seen and easily felt. The gland varies in size from time to time, especially during menstruation.

Case VIII. (Photo 7.) Negro man with a symmetrically developing diffuse parenchymatous goiter. The goiter has grown pace for pace, always keeping the form and the proportions of the normal thyroid gland. This is the most remarkable case of symmetrical hypertrophy in my experience.

Case IX. (Photos 5 and 6.) Middle aged woman, with a very large colloid goiter, removed at two seances, one using general anaesthesia and the other, under cocaine. The patient is pictured after the first operation immediately after removing the stitches on the eighth day. Photo 5 shows the right lobe removed and split open; most of the bulk is colloid. The surface also shows areas of dense calcarious deposits.

Case X. (Photo 11.) A young woman with a typical case of exaphthalmic goiter—exaphthalmos, goiter and tachycardia—came to me as she appears in the photograph. She had been operated on by another surgeon, who removed one lobe and tied off the thyroid vessels on the opposite side. The symptoms still persisted after operation. The patient must undergo removal of the old scar and lobectomy on the other side. (The first operation was in no wise a failure; the surgical principle applied was good and still holds good; that is, the reduction of the over-secreting surface of the thyroid gland. The amount to remove is a difficult point to determine; ¾ may be removed with impunity.)

Case XI. (Photo 17.) A young girl, eleven years old, not yet arrived at the age of puberty. The case shows exophthalmos tachycardia (pulse-rate 170), small but visible goiter. These symptoms have been present for about two years. The case has just been seen and the following treatment prescribed—rest in bed, hydrobromate of quinine, bowels kept open with sodium phosphate and ice-bag over the gland. In a few weeks ligation of the superior thyroid vessels and subsequently thyroidectomy on one side.

Case XII. (Photo 18.) A young woman, fifteen years old, puberty at eleven. There was present a large goiter, exophthalmos, tachycardia (rate 108). The picture shows the case on the tenth day after the removal of the right lobe and isthmus—about three-quarters of the entire gland. The pulse is now about 70. The specimen of the lobe removed and another from an almost parallel case are shown in photo 19.

Case XIII. (Photo 12.) A young man with a moderate sized goiter, tachycardia (pulse rate 140), and very slight or no exophthalmos. The case is not being benefited by medical treatment. Thyroidectomy is projected.

Case XIV. (Photo 13.) A young colored woman, with a small goiter, slight exophthalmos and eye symptoms and tachycardia (pulse rate 100). Patient improved under medical treatment: Rest, hydrobromate of quinine, atrophine, and sodium phosphate.

Case XV. (Photo 14.) Middle aged woman, who had a small goiter, exophthalmos, and tachycardia. These symptoms disappeared on removal of broken down tonsils and disarticulation of a leg for osteo myelitis. (This case illustrates a class of cases secondary to some primary toxemia or infection.)

Case XVII. (Photo 8.) A middle aged woman with a nodular goiter of the colloidal type and forming a large mass of conglomerated follicles confined to one lobe of the thyroid gland. There is nothing remarkable about this case, except the presence of the goiter.

Case XVIII. (Photos 9 and 10.) A young man with a true cyst and a surrounding adenomatosis of the right lobe of the thyroid gland. Gave history of an already existing tumor in the same location before a fall on the neck, when the tumor suddenly increased in size. The swelling decreased somewhat, then remained about constant. Patient also complained of general nervousness, puffy face and a fast heart. These symptoms all disappeared after a lobectomy on the right side. (Is this one of the non-hyperplastic toxic goiters described by Wilson (5) and Plummer (6) as distinguished from hyperplastic toxic goiter, that is exophthalmic goiter?) Pathologically the case is one of a hemorrhage occurring in an existing adenoma, giving rise to a cyst lined by epithelium, a true cyst; the cyst wall is surrounded by thyroid tissue considerably increased in bulk and presenting the microscopical picture of an adenomatosis (photo 10).

Case XIX. (Photo 20.) A girl, eighteen years old, with a very short linear scar along the medial border of the sternomastoid muscle. Through this small incision a cyst of the right lobe of the thyroid gland was removed as follows: The lobe over the cystic portion was exposed, the gland opened and the capsule of the cyst separated from the gland in front;

13
14
15
16
17
18

the cyst itself, then emptied of its contents, and the collapsed capsule easily separated from the surrounding gland. (Even solid isolated tumors, when they are encapsulated, can be collapsed by hollowing out the contents from the center outward and curetting the inner lining of the capsule. This twisted up capsule makes a very small bundle and can be readily drawn out through a small skin incision. Young women think a great deal about the after appearance of their necks and too often this factor alone will determine the operation.)

Case XX. (Photos 15 and 16.) A woman just beyond middle age with a simple goiter of almost exactly the same clinical appearance as the case from which the goiter pictured in photo 16 was removed. This latter was rapidly growing, producing great difficulty in swallowing and hoarseness of the voice. The gland had broken through the skin under pus formation several times. At operation the gland was found adherent to all the surroundings and was very difficult to liberate with sharp dissection. After removing the mass, which served as a veritable hame over the front of the neck, the internal jugular veins dilated extremely, so much so that I could not control the dilatation, and the patient died on the table of shock, shortly after the termination of the operation. This is the only death from operation in all my series of goiters, from which these twenty cases have been chosen as types.

## Figures and Tables

**Figure f1:**
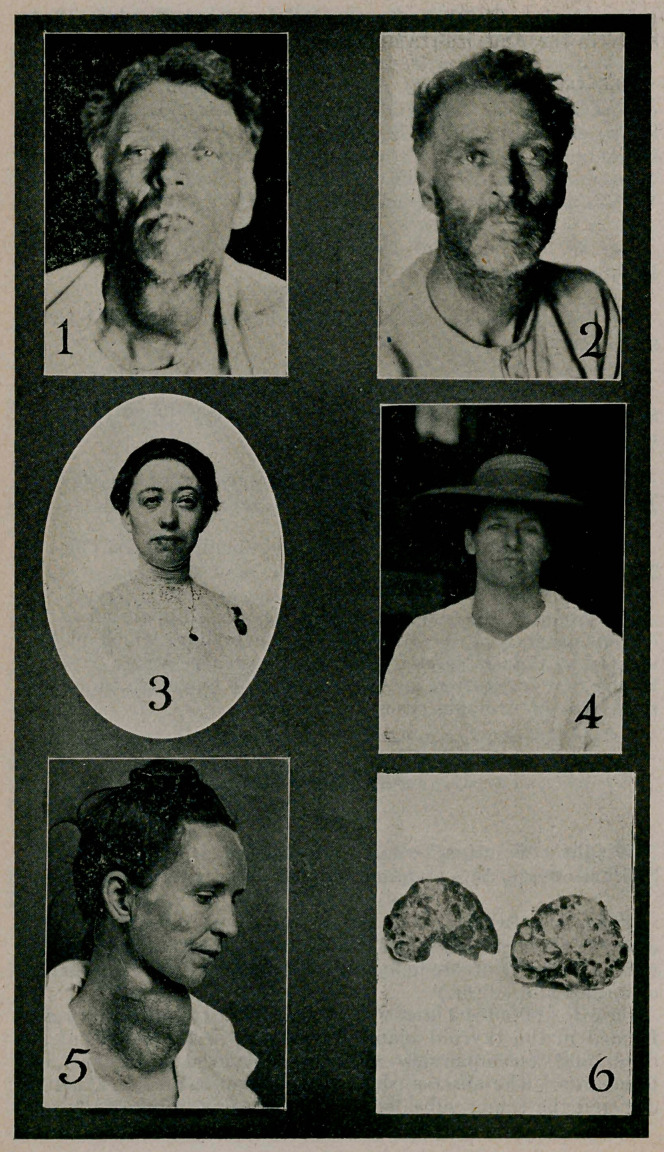


**Figure f2:**
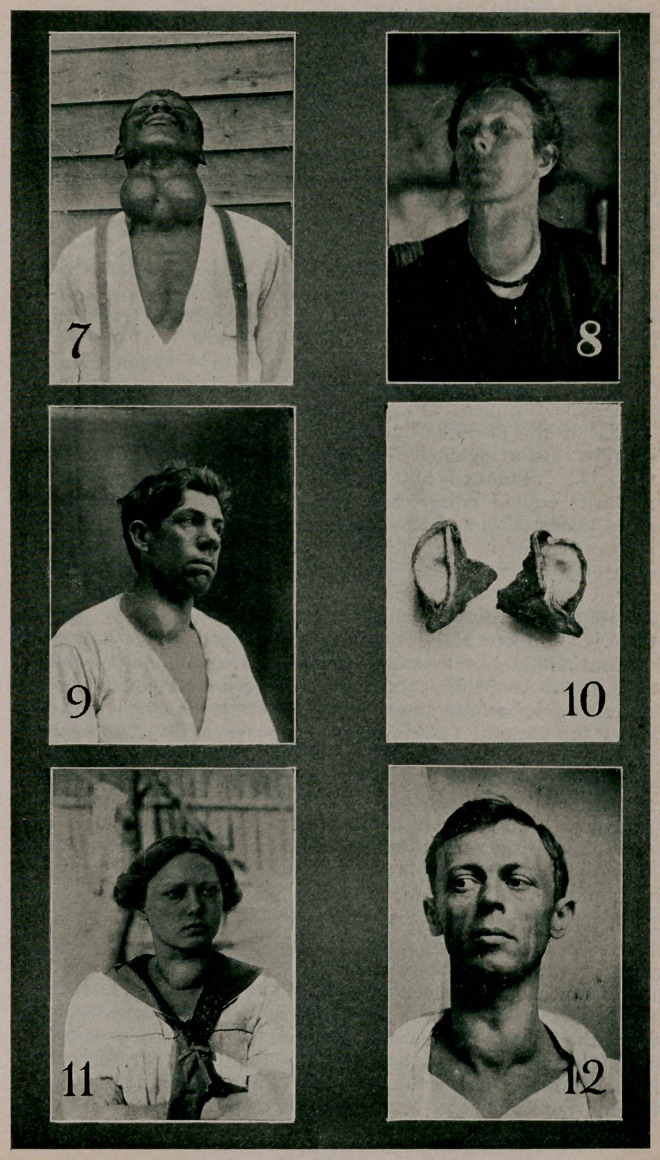


**Figure f3:**
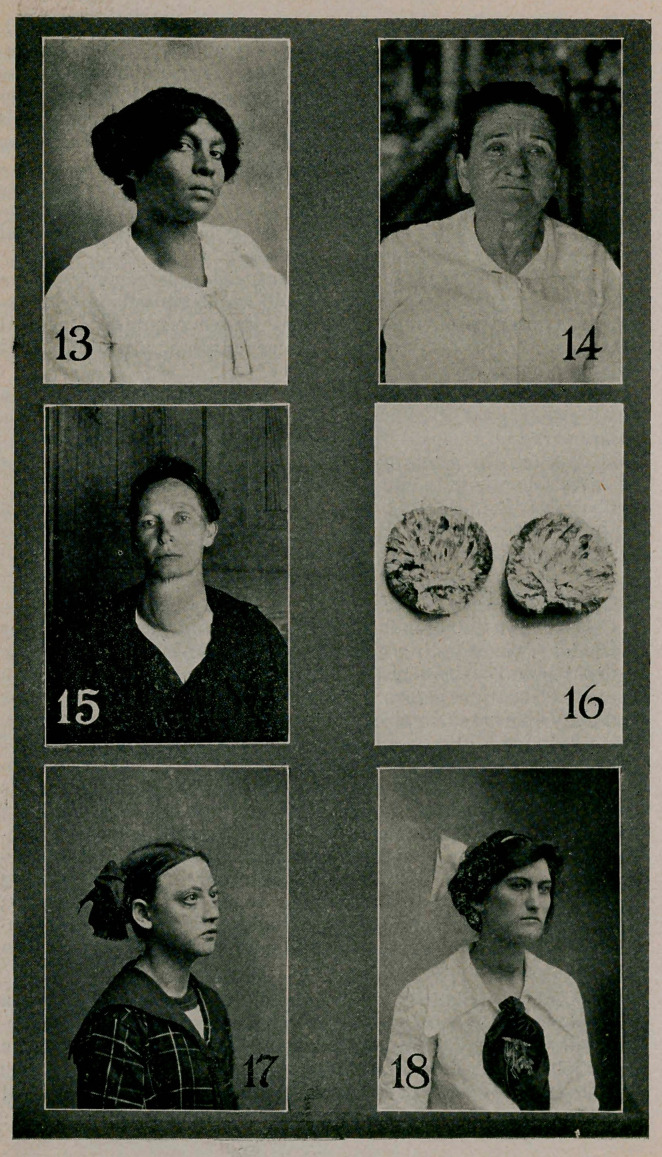


**Figure f4:**
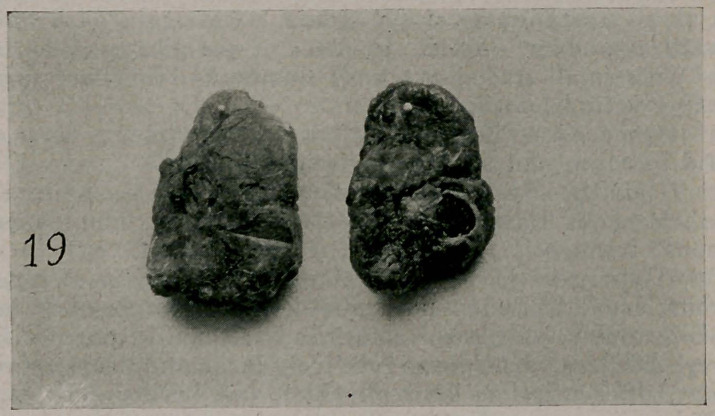


**Figure f5:**